# Management of Melanoma Brain Metastases in the Era of Targeted Therapy

**DOI:** 10.1155/2011/845863

**Published:** 2011-12-15

**Authors:** Daniela Gonsalves Shapiro, Wolfram E. Samlowski

**Affiliations:** ^1^Section of Melanoma, Renal Cancer and Immunotherapy, Nevada Cancer Institute, One Breakthrough Way, Las Vegas, NV 89135, USA; ^2^Comprehensive Cancer Centers of Nevada, Henderson, NV 89014, USA; ^3^US Oncology Research Developmental Therapeutics and Genitourinary Committees, Woodloch, TX, USA; ^4^University of Nevada, Reno, NV, USA

## Abstract

Disseminated metastatic disease, including brain metastases, is commonly encountered in malignant melanoma. The classical treatment approach for melanoma brain metastases has been neurosurgical resection followed by whole brain radiotherapy. Traditionally, if lesions were either too numerous or surgical intervention would cause substantial neurologic deficits, patients were either treated with whole brain radiotherapy or referred to hospice and supportive care. Chemotherapy has not proven effective in treating brain metastases. Improvements in surgery, radiosurgery, and new drug discoveries have provided a wider range of treatment options. Additionally, recently discovered mutations in the melanoma genome have led to the development of “targeted therapy.” These vastly improved options are resulting in novel treatment paradigms for approaching melanoma brain metastases in patients with and without systemic metastatic disease. It is therefore likely that improved survival can currently be achieved in at least a subset of melanoma patients with brain metastases.

## 1. Introduction

 It is estimated that metastatic melanoma was responsible for more than 8700 caner-related deaths in the United States in 2010 [1]. Melanoma ranks fourth in the incidence of brain metastases, behind lung, breast, and unknown primary cancers [2]. In addition, metastatic melanoma patients overall have a median survival of only 6–10 months and a 5-year survival of less than 10% [3]. There has been virtually no improvement in survival of those patients in the past several decades [3]. The trend toward targeted therapies [4], novel immunotherapeutic agents [5], stereotactic radiosurgery, and improved neurosurgical interventions give great hope to improving this trend in the coming years.

## 2. Screening

 In our own institutional experience, the risk of brain metastasis in malignant melanoma is approximately 30% at presentation of metastatic disease and may rise to 60% over the next two years in surviving patients [6]. This risk increases substantially with disease duration, as up to 75% of Stage IV melanoma patients are found to have brain metastases at autopsy [7–10]. The implication of this finding is that brain metastases are an almost inevitable part of the disease process, if patients survive long enough. Therefore, the potential development of brain metastases needs to be anticipated in both staging and follow-up strategies. Further evidence of the high risk of brain metastases even in earlier stage disease can be drawn from the recently completed Southwest Oncology Group S0008 adjuvant therapy study. In these Stage IIIb and IIIc patients, there was a 16% isolated CNS failure rate within the first 2 years as the initial site of relapse (Samlowski et al., manuscript in preparation). Even with such a high and alarming incidence, no standard screening recommendations currently exist for Stage III or IV melanoma patients to detect presymptomatic disease. This is in part because of the increasingly outdated perception that brain metastases represent a terminal event. This has discouraged physicians from attempts at early detection.

 Patients presenting with neurologic symptoms, such as seizure or hemiplegia, are commonly found to have either large (greater than 4 cm) or numerous (greater than 7) lesions. These clinical presentations are very difficult to treat and generally become palliative situations.

 In contrast to this, less numerous and smaller (<2 cm) lesions encountered in asymptomatic patients are much more readily and effectively treated. It has been shown that MRI scans with gadolinium contrast are superior to CT scans when investigating for brain metastases [11]. Unfortunately, there are no randomized clinical trials, to date, to define the optimal screening strategy (e.g., timing of scans and duration of followup) that would lead to efficient and cost-effective detection. Another caveat is a need for these studies to demonstrate that earlier detection actually leads to improved functional outcome and survival, rather than apparent differences induced by lead-time bias. With current improvements in therapy, it is time, in our opinion, to identify high-risk patients and begin such studies.

## 3. Palliative Therapy

 When dealing with metastatic brain lesion, neurologic symptoms often present suddenly, including devastating headaches, dizziness, and seizures. This may be due to the development of peritumoral edema or bleeding into previously silent metastases. The initial treatment is generally oral or intravenous glucocorticoids. Their anti-inflammatory activity helps to reduce peritumoral edema and swelling and prevent further neurological deficits [12]. Antiepileptic drugs are indicated for treatment of patients who have experienced seizures secondary to brain metastasis [13]. However, studies have shown no benefit in prophylactic treatment of patients with antiepileptic medications. These medications can produce both side effects and potential interactions with chemotherapeutic agents [14].

## 4. Surgical Resection

 Treatment of brain metastases has historically been based on the use of surgical resection, when possible. The consideration of surgical resection is dependent upon the number of lesions, the overall state of systemic disease and symptoms at the time of diagnosis. In general, patients with minimal or no systemic disease, one or two superficial metastases, and excellent functional capacity will benefit from surgery. In this very small group of patients (perhaps 5% overall), surgical intervention has been shown to improve quality of life, as well as survival [10, 15]. Factors adversely affecting the decision to perform surgery include the size of the tumor (greater than 2 cm) and the location, (the ability to resect lesions without significant neurologic sequelae) [16, 17]. This surgical decision-making process is aided by current technology, using stereotactic localization of the lesion at the time of surgery [15]. Additionally, intraoperative functional mapping may help to decrease the risk of neurologic sequelae at the time of resection [18, 19]. It should be noted that surgical resection may also benefit the more severely symptomatic patients, as excision of lesions causing significant edema or herniation can have a valuable palliative effect. After surgery, postoperative WBRT has been shown to lead to longer functional independence for the patients, with decreasing the risk of relapsing at the CNS resection site [20]. 

## 5. Whole Brain Radiotherapy (WBRT)

 Traditionally, whole brain radiotherapy (e.g., 30 Gy in 10 fractions over 2 weeks elapsed time) has become a *de facto* (but not evidence-based) standard treatment for brain metastasis. Some patients may have significant palliation of symptoms following treatment. Melanoma has traditionally been considered radioresistant to these treatment doses, however [21]. A number of series have established a median survival of 3-4 months. For example, a large recent series from the Sydney Melanoma Unit, encompassing close to 700 patients, showed that resected patients, with or without WBRT had a median survival of 8.7 to 8.9 months, where as patients treated solely with WBRT had a median survival of only 3.4 months. This is clearly a poor outcome, although the patient selection process for surgery versus radiotherapy was not described. As has been seen with all treatment modalities, a patient with excellent performance status and no systemic metastatic disease, and/or a single lesion relapse, WBRT have a somewhat better outcome (termed Recursive Partition Class 1) [22]. In reality, these patients are very uncommon in a medical oncology clinic, where the vast majority of patients have active systemic metastatic disease (Class II or III). Surgery may add survival benefit in selected WBRT-treated patients, such as those with a solitary brain metastasis [23, 24].

## 6. Radiosurgery 

 In contrast to large (>4 cm) lesions, smaller metastatic lesions have become easily treated via radiosurgery. In these situations solitary or multiple lesions can be treated with either stereotactic radiosurgery (SRS) or Gamma-knife (GK) treatment. At present, no head-to-head comparisons exists randomizing patient to SRS versus GK. Based on radiobiology studies, it is currently believed that patient outcomes should be comparable between these approaches. The major benefit of radiosurgery is that it allows for treatment of brain lesions that would otherwise be inoperable, including lesions in deep structures and close to functionally critical brain structures. This can be accomplished due to the rapid drop-off of radiation dose at the margin of the treatment volume and the capability of computerized MRI-based dosimetry planning. In small case series local control, survival following radiosurgery (either SRS or GK) has been quite encouraging [25–39].

 As with all modalities there are preferred parameters such as lesion size (ideally under 3 cm) and a total number of lesions (7 or less) that make radiosurgery most effective. The maximum number of lesions that can be effectively treated is still evolving, but is generally functionally defined as the number that can be effectively treated in about 1 hour of patient immobilization. There may be an important biological issue, to consider as well, as patients with oligometastatic disease (1–3 metastases) may have a different biology and outcome to those with many (10–20) metastases. The optimal break point in deciding whether to employ radiosurgery or WBRT as the primary treatment modality remains to be better defined as radiosurgery technology evolves to allow more rapid treatment of increasing numbers of lesions. 

## 7. Should WBRT Automatically Be Added after SRS?

 Historically, WBRT was used to treat brain metastases and after the development of SRS/GK, these were initially added to boost the radiation dose to larger lesions, which were unlikely to be controlled by WBRT alone. It soon became apparent that primary SRS/GK of smaller lesions provided excellent long-term lesion control as a primary treatment, and that many of these patients did not appear to require additional CNS therapy (either WBRT or surgery) [40]. This has led to an ongoing debate about whether GK/SRS should be followed by immediate WBRT for treatment of patients with melanoma brain metastases, or whether WBRT could be deferred. There have now been four randomized clinical trials including patients with 1–3 brain metastases from a variety of cancers. These studies have generally shown that local control at the SRS-/GK-treated site is not improved by WBRT, but that the development of new brain metastases is significantly decreased by immediate addition of WBRT [38, 41–43]. Overall survival does not appear to have been affected by immediate WBRT. This is because delayed salvage with additional SRS/GK [44, 45], or delayed WBRT, is possible (in the <50% of patients who progress in the CNS) [46]. Thus, quite a few investigators have concluded that delaying WBRT may be appropriate for some patients, with the caveat that these patients need to be followed closely with the intent of early retreatment at CNS progression [46, 47]. This has the potential to decrease neuropsychiatric sequelae and memory loss from radiotherapy in the increasing number of long-term survivors of brain metastases [48], as in our experience 50% of patients never require salvage therapy [6]. In addition, the only patients who developed radiation necrosis as a treatment complication in our series had received both SRS and WBRT [6].

## 8. Systemic Therapy

 Systemic therapy has historically not been effective in melanoma therapy and, therefore, has rarely been utilized as primary treatment for metastatic brain lesions [49]. However, most patients with brain metastases also have active systemic metastases. Once brain metastases are controlled (e.g., with surgery, or SRS/GK), failure to treat systemic disease results in invariable progression and death of the patient (both due to reseeding of the CNS and systemic progression). This was particularly true in the era of ineffective chemotherapy. Remarkably, if even modestly effective IL-2-based immunotherapy was added after control of CNS metastases, prolonged survival appeared to be achievable in some patients. We initially demonstrated this principle by using biochemotherapy after SRS treatment of brain metastases and showed that median survival of over 1 year could be achieved, with 15% of patients alive at 3 years [50]. This observation is currently being recapitulated in clinical trials of the CTLA-4 monoclonal antibody ipilimumab. In these studies, patients with SRS/GK controlled brain metastases have been included and have shown significant survival prolongation. These trials have included patients with apparent CNS responses to ipilimumab therapy [5, 51, 52]. What appears most interesting is that if the CNS lesions can be effectively controlled for an extended period (perhaps 18–24 months), eventual long-term CNS control appears to be achievable, as was previously seen with biochemotherapy [50].

## 9. Targeted Therapies

 A series of somatic genetic mutations has been identified in melanoma cells, leading to the research in specific targeted therapies. These include mutations in NRAS, BRAF, C-KIT (in cutaneous melanoma) [53], GNAQ, and GNA11 (in ocular melanoma) [54, 55], as well as in tumor suppressor genes such as PTEN and p16 [56]. A single mutation in BRAF (V600E) is present in about 50–60% of human cutaneous melanomas [57]. This substitution triggers a kinase-signaling cascade that can lead to cell growth, tissue invasion, and ultimately metastasis [57, 58]. While inhibitors of the wild-type B-RAF are not clinically active in melanoma [59], inhibitors of the V600E mutation are highly active in patients whose tumor expresses this mutation [4].

 Unfortunately, most early B-RAF V600E inhibitor trials were designed to specifically exclude patients with brain metastases. In October of 2010, at the 35th Congress of the European Society for Medical Oncology (ESMO), a phase I/II clinical trial involving the newly developed GSK2118436 V600E BRAF inhibitor was presented. This trial allowed enrollment of a cohort of 10 patients with V600 BRAF positive melanomas and brain metastases. All patients experienced control of their brain metastases, with 90% experiencing a significant decrease in lesions size. This response was seen in brain metastases measuring 3 mm or larger in diameter, with an estimated reduction of between 20 and 100%. This is the first targeted agent that has shown objective responses in the CNS. This observation will be followed up with larger clinical trials in the United States and internationally.

## 10. Conclusions

 Treatment of brain lesions in metastatic melanoma patients remains an ongoing challenge. As the incidence of melanoma has increased, so has the need to confront this problem. We have begun to rethink our approaches, including the screening of appropriate patient populations (overviewed in Table 1). There have been refinements in traditional modalities, such as neurosurgery, as well as development of stereotactic guidance and functional mapping techniques that have enhanced their effectiveness. The evolution of computer-image-guided radiosurgery has further improved treatment options in radioresistant cancers and limited collateral injury to normal tissue structures. Effective sequential or concurrent therapy of CNS metastases and systemic disease has proven to be possible, especially with the development of new and active immunotherapy and targeted therapeutic agents. There remains more work to be done, especially with improving local CNS control at the treated sites (the eventual cause of about 1/3 of mortality), development of new CNS metastases (resulting in another 1/3 of patient deaths), and systemic treatment failures (the remaining 1/3 of patient deaths). Close followup of treated patients is essential, as retreatment of the CNS is frequently possible, with long-term salvage as a potential goal in some patients. With the continuing evolution of therapy and continued clinical trials, hope of an entire new spectrum of therapeutic options is within our grasp. We must remain steadfast in our pursuit of these attainable goals through continued high-quality patient-centered research.

## Figures and Tables

**Table 1:** Potential management strategies for melanoma patients with brain metastases.

Brain metastases	Largest lesion	Symptomatic*	Suggested CNS treatment	Systemic metastases	Systemic therapy^§^
1	<3 cm	yes or no	surgery^†^GK or SRS^•^	no	not suggested
1	>4 cm	yes or no	surgery^†^	no	not suggested
2–5	<4 cm	yes or no	GK or SRS	no	no
2–5	<4 cm	yes or no	GK or SRS	yes	yes^¶^
>5	<4 cm	yes or no	WBRT^¥^	no	no
>5	<4 cm	yes or no	WBRT^¥^	yes	yes^¶^

*Palliative glucocorticosteroid administration should be considered to decrease symptomatic edema, if present.

^†^Resectability may depend on location related to critical brain structures.

^•^GK and SRS are probably equivalent to surgical resection for lesion control if <2 cm.

^§^The majority of these patients do not progress with systemic disease and there is little evidence that early systemic treatment improves either the risk of systemic relapse or helps control CNS metastases.

^¶^Patients should have CNS lesions treated and controlled first, potentially effective agents include immunotherapy (ipilimumab, possibly IL-2) and targeted therapy (B-RAF inhibitor, etc.), if the appropriate activating mutation is present in tumors.

^¥^Stereotactic boost to dominant lesions > 1 cm after WBRT may increase local lesion control and survival in patients with early CNS control and controlled systemic disease based on randomized trials.
